# Fear of progression and association factors in stroke patients: a latent profile analysis

**DOI:** 10.3389/fpsyg.2026.1741344

**Published:** 2026-03-03

**Authors:** Yunyun Liu, Xiangrui Li, Ting Zhao, Bo Wan, Leyao Han, Yaya Xi, Jingying Xiong, Meishan Zhang, Yingqiao Wang, Xinman Dou, Weiping Li, Xinglei Wang

**Affiliations:** 1 School of Nursing, Lanzhou University, Lanzhou, China; 2 The Second Hospital & Clinical Medical School, Lanzhou University, Lanzhou, China; 3 Department of Cardiovascular Medicine, Lanzhou University Second Hospital, Lanzhou, China; 4 No.988 Hospital of Joint Logistic Support Force, Zhengzhou, China; 5 Department of Nursing, Lanzhou University Second Hospital, Lanzhou, China; 6 The Armed Police Forces Hospital of Gansu, Lanzhou, China

**Keywords:** cerebrovascular accidents, fear of progression, latent profile analysis, risk factors, risk perception, stroke patients

## Abstract

**Background:**

Fear of progression (FoP) is a prevalent psychological issue among stroke patients. Previous studies failing to distinguish characteristics of patient groups with varying FoP levels. Latent profile analysis (LPA) classifies individuals into distinct subgroups via continuous FoP indicators, boosting classification accuracy by accounting for variable uncertainty. Given FoP’s heterogeneity, investigating FoP profiles and their influencing factors in stroke patients is clinically significant for personalized psychological care and improved patient quality of life.

**Methods:**

A total of 366 stroke patients were selected as study subjects through convenience sampling, and a cross-sectional survey was conducted. FoP was assessed using the Fear of Progression Questionnaire-Short Form (FoP-Q-SF, 2 dimensions, 12 items). Independent variables included demographic characteristics, clinical indicators, the Recurrence Risk Perception Scale for Stroke patients (RRPSS), and the Medical Coping Modes Questionnaire (MCMQ). LPA was performed on the FoP-Q-SF items to identify subgroups. The R3STEP method was used to analyze influencing factors of subgroup membership, and the BCH method was applied to compare differences in distal outcomes across subgroups. Statistical significance was set at *p* < 0.05.

**Results:**

The study sample had a mean age of 63.93 ± 10.58 years, with 70.5% males and 65.0% first-ever stroke patients. Two latent profiles were identified: Low-FoP Adaptive Type (C1, 48.6%) and High-FoP Sustained Type (C2, 51.4%). The R3STEP showed that age 18–59 years (OR = 0.476, 95%CI = 0.245–0.924, *p* = 0.028), hypertension comorbidity (OR = 0.402, 95%CI = 0.237–0.683, *p* = 0.001), higher RRPSS score (OR = 0.971, 95%CI = 0.946–0.995, *p* = 0.022), MCMQ-confrontation (OR = 0.920, 95%CI = 0.863–0.982, *p* = 0.011), and MCMQ-avoidance (OR = 0.796, 95%CI = 0.723–0.876, *p* < 0.001) were significant influencing factors (all *p* < 0.05). BCH analysis indicated that C2 patients had higher RRPSS score (*p* < 0.001), higher NIHSS score (*p* = 0.002) and lower adaptive coping ability than C1.

**Conclusion:**

This study revealed significant heterogeneity in FoP among stroke patients. Age, hypertension comorbidity, excessive recurrence risk perception, MCMQ-confrontation, and MCMQ-avoidance were associated with high FoP. Healthcare providers should prioritize identifying high-risk individuals and develop tailored interventions to reduce FoP and improve rehabilitation outcomes.

## 1 Introduction

Globally, stroke ranks as the second leading cause of mortality (11.6% of total deaths) and the third leading cause of disability (5.7% of total disability-adjusted life-years; Feigin et al., 2021). It is also the primary cause of mortality and disability in adults in China (Ma et al., 2021). Studies have shown recurrence risks of 3.54 to 24.50% within the first year following an index stroke (Pu et al., 2018). This rises to 9.40 to 22.90% over 5 years (Kumral et al., 2015). Approximately 80% of survivors experience varying degrees of functional impairment (Stinear et al., 2020), and over 33% of patients develop depression (Liu et al., 2023). The significant incidence and disability rates of stroke severely impact patients’ well-being and generate substantial burdens for families and society. During rehabilitation, patients often face ongoing concerns about disease recurrence, functional decline, and loss of social roles, leading to a pervasive psychological fear known as “Fear of Progression (FoP)” (Ferro and Santos, 2020).

FoP refers to an individual’s fear of potential disease recurrence, deterioration, or progression, encompassing concerns about the physiological, psychological, and social consequences of disease development, as well as a general fear of threats to life and health (Zhang et al., 2023). Among stroke patients, over 50% fear stroke recurrence (Choi and Lee, 2017), particularly the potential for death or severe disability. Transient, moderate fear can motivate positive health behaviors (Lebel et al., 2013), but persistent or excessive FoP may impair physical-functional recovery, mental health, and coping strategies (Reb et al., 2020). This can delay rehabilitation, increase the risk of recurrence, and ultimately adversely affect disease prognosis, quality of life, and medical resource utilization (Lebel et al., 2013). Notably, an Asian study explained, stroke-related disability is closely associated with psychological distress, which inadvertently exacerbates FoP (Weerasekara et al., 2024). This highlights the urgency of addressing psycho-social aspects in stroke rehabilitation.

Latent profile analysis (LPA) is a person-centered analysis that focuses on the heterogeneity of individual responses by classifying individuals into distinct subgroups with common profiles (Marsh et al., 2009). However, existing research on FoP has predominantly focused on patients with cancer and cardiac disorders (Schapira et al., 2022; Wang X. et al., 2024), while studies on FoP in patients with stroke remain scarce. Furthermore, previous studies often treated patients as a homogeneous group, using scale scores to reflect fear levels. This study implements LPA to stratify FoP subgroups among stroke survivors and elucidate the contributing determinants, thereby generating evidence-based guidelines for precision clinical interventions.

## 2 Method

### 2.1 Participants

This study enrolled 366 patients with stroke from three tertiary care hospitals in Gansu Province, China. The 300–500 participant range was derived from LPA-based sample size calculation protocols (Ferguson et al., 2020). The inclusion criteria for participants were as follows: (a) age ≥ 18 years, (b) confirmed by computed tomography or magnetic resonance imaging, (c) participants were conscious and capable of completing the questionnaire independently, (d) all participants provided voluntary written informed consent. The exclusion criteria were as follows: (a) patients with severe comorbidities affecting major organ systems (e.g., cardiac, pulmonary, or renal), (b) patients currently enrolled in other clinical studies.

### 2.2 Measures

The demographic questionnaire covered age, gender, occupation, marital status, residence area, education, and monthly income.

The National Institutes of Health Stroke Scale (NIHSS) is clinically employed to assess neurological deficits and track progression in stroke patients (Runde, 2020). Scores range from 0 to 45, with higher values indicating more severe impairment. Stroke was classified as mild (0–4), moderate (5–15), or severe (> 15).

The Fear of Progression Questionnaire-Short Form (FoP-Q-SF) evaluates the fear of disease progression (Mahendran et al., 2020). The Chinese version of this scale, which was sinicized and revised by Wu et al. (2015), comprises two dimensions (physical health and social-family) and includes 12 items. Responses are measured on a 5-point Likert scale (1 = never, 5 = always). Total scores range from 12 to 60, with higher scores reflecting a greater FoP. A cutoff score of ≥ 34 indicates clinically significant psychological dysfunction. The Cronbach’s *α* coefficient for the total scale is 0.883, while the coefficients for the physical health and social-family dimensions are 0.829 and 0.812, respectively. This scale demonstrates robust reliability and validity and has been extensively utilized in China.

The Recurrence Risk Perception Scale for Patients with Stroke (RRPSS) was developed by Lin et al. (2021a) (Chinese version, Cronbach’s *α* = 0.850). It includes two sections: Section 1 evaluates subjective recurrence likelihood, and Section 2 (17 items, 3 dimensions) assesses recurrence-related concerns. Scores range from 18 to 76, with higher values indicating greater perceived recurrence risk.

The Medical Coping Modes Questionnaire (MCMQ) measures coping styles in stroke patients (Shen and Jiang, 2000). This 20-item version covers three dimensions: confrontation, avoidance, and resignation (4-point Likert scale). In this study, Cronbach’s *α* coefficients were 0.64 (confrontation), 0.85 (avoidance), and 0.67 (resignation). Widely applied in clinical research to evaluate patients’ coping strategies, this scale still boasts extensive application value in coping style assessment despite the fact that some dimensional coefficients failed to reach the ideal standard.

### 2.3 Procedures

Our study was approved by the Ethics Committee of Lanzhou University Second Hospital (Approval No. 2025A-658), administrative clearance was obtained from the three participating hospitals prior to data collection, and written informed consent was obtained from all patients. A pretest was conducted in 20 stroke patients to evaluate the clarity and feasibility of the questionnaires. Based on pretest feedback, two ambiguous items in the RRPSS were revised (e.g., “I worry about recurrence” was clarified to “I often worry about stroke recurrence in daily life”). Investigators (3 nursing graduate students) received standardized training on questionnaire administration, including verbal administration skills, confidentiality principles, and on-site verification procedures, with a training pass rate of 100%. For individuals with limited literacy (education level ≤ primary school) or elderly participants (age ≥75 years), items were verbally administered item-by-item by trained investigators. On-site verification was conducted immediately after questionnaire completion to ensure no missing or contradictory responses. All participant responses were de-identified to ensure confidentiality.

### 2.4 Data analysis

We analyzed participants’ demographic characteristics using descriptive statistics. LPA was conducted using Mplus 8.3 software with the following technical parameters: (1) Estimator: Robust maximum likelihood (MLR); (2) Convergence criteria: Maximum iterations = 10,000; (3) Random starts: 500 initial stage starts, 100 final stage starts; (4) Optimization: Multiple group optimization; (5) Local maxima check: Solutions from 10 independent random start sets were compared to ensure the global maximum likelihood solution was obtained. Manifest indicators were the 12 items of the FoP-Q-SF. Model fit was evaluated using Akaike Information Criterion (AIC), Bayesian Information Criterion (BIC), Adjusted Bayesian Information Criterion (ABIC), Entropy, Lo–Mendell–Rubin (LMR) test, and Bootstrap Likelihood Ratio Test (BLRT). The optimal class number was determined by: (a) lower AIC/BIC/ABIC; (b) Entropy > 0.80; (c) significant LMR/BLRT (*p* < 0.05) indicated a superior fit of the K class model over the K-1 class model (Tein et al., 2013).

After identifying latent profiles: (1) Univariate analyzes: Chi-square tests and ANOVA were used to compare differences between subgroups; (2) Influencing factors: R3STEP method was used to assess the association between independent variables and subgroup membership (reference group: C1); (3) Distal outcome comparison: BCH method was applied to compare differences in NIHSS, RRPSS, and MCMQ scores across subgroups (controlling for classification error).

Statistical significance was set at *p* < 0.05. LPA was conducted in Mplus 8.3, and other analyzes were performed using SPSS 27.0.

## 3 Results

### 3.1 Demographic and clinical characteristics

Our study initially surveyed 370 stroke patients, after excluding 4 invalid questionnaires, the final analyzed sample comprised 366 cases, yielding a valid response rate of 99%. The mean age of the sample was 63.93 ± 10.58 years. This study included 258 males (70.5%), 148 workers/farmers (40.4%), 44 patients (12.0%) living alone, and 126 patients (34.4%) with primary school education or below.

Disease-related clinical data of the patients: 238 patients (65.0%) experienced first-ever strokes, 179 patients (48.9%) had multiple lesions, 257 (70.2%) were comorbid with hypertension, 125 (34.3%) with diabetes mellitus, and 60 (16.4%) with cardiovascular diseases. Functional assessments revealed that 67 patients (17.9%) had moderate-to-severe dependence on self-care abilities, and 91 (24.9%) were classified as having moderate-to-severe strokes based on NIHSS scores. The mean scale scores were as follows: FoP-Q-SF (32.67 ± 11.84), RRPSS (51.62 ± 10.43), and MCMQ (38.84 ± 5.96). The results are shown in Table 1.

**Table 1:** General and clinical characteristics of sample [*N* = 366, n (%)].

Variables	Class 1 n(%)	Class 2 n(%)	χ^2^ /F	*p*
Age(years)				
18–59	47(26.4)	71(37.8)	5.402^a^	0.020^*^
≥60	131(73.6)	117(62.2)		
Gender				
Male	138(77.5)	120(63.8)	8.648^a^	0.004^*^
Female	40(22.5)	68(36.2)		
Occupation				
Employed	15(8.4)	20(10.6)	7.675^a^	0.104
Retired	77(43.3)	57(30.3)		
Unemployed	9(5.1)	15(8)		
Worker/Farmer	64(36.0)	84(44.7)		
Freelancer	13(7.3)	12(6.4)		
Marital status				
Single	3(1.7)	4(2.1)	1.469^a^	0.689
Married	165(92.7)	170(90.4)		
Divorced	2(1.1)	1(0.5)		
Widowed	8(4.5)	13(6.9)		
Living arrangement				
Living alone	22(12.4)	22(11.7)	0.037^a^	0.847
Living with others	156(87.6)	166(88.3)		
Residential area				
Rural	59(33.1)	78(41.5)	2.849^a^	0.241
Town	31(17.4)	31(16.5)		
Urban	88(49.4)	79(42)		
Primary caregiver				
Spouse	117(65.7)	106(56.4)	7.631^a^	0.106
Parents	2(1.1)	11(5.9)		
Children	39(21.9)	47(25.0)		
Employees	1(0.6)	1(0.5)		
Other	19(10.7)	23(12.2)		
Educational background				
Primary school or below	54(30.3)	72(38.3)	4.362^a^	0.225
Middle school	57(32.0)	60(31.9)		
High school	46(25.8)	43(22.9)		
University or above	21(11.8)	13(6.9)		
Household monthly income(CNY)				
<2000	57(32.0)	82(43.6)	8.200^a^	0.042^*^
2000–4,999	85(47.8)	84(44.7)		
5,000–9,999	35(19.7)	22(11.7)		
>10,000	1(0.6)	0(0)		
Number of stroke episodes				
First episode	118(66.3)	120(63.8)	0.244^a^	0.622
Recurrence	60(33.7)	68(36.2)		
Number of lesions				
Single lesion	103(57.9)	84(44.7)	6.360^a^	0.012^*^
Multiple lesions	75(42.1)	104(55.3)		
Hypertension comorbidity				
No	69(38.8)	40(21.3)	13.371^a^	<0.001
Yes	109(61.2)	148(78.7)		
Diabetes comorbidity				
No	123(69.1)	118(62.8)	1.632^a^	0.201
Yes	55(30.9)	70(37.2)		
Cardiovascular comorbidity				
No	155(87.1)	151(80.3)	3.048^a^	0.081
Yes	23(12.9)	37(19.7)		
Other chronic comorbidities				
No	162(91)	167(88.8)	0.479^a^	0.489
Yes	16(9.0)	21(11.2)		
Smoking				
Yes	81(45.5)	70(37.2)	2.581^a^	0.108
No	97(54.5)	118(62.8)		
Alcohol drinking				
Yes	51(28.7)	44(23.4)	1.310^a^	0.252
No	127(71.3)	144(76.6)		
ADL				
100	26(14.6)	19(10.1)	10.106^a^	0.018^*^
61–99	130(73.0)	124(66.0)		
41–60	16(9.0)	26(13.8)		
≤40	6(3.4)	19(10.1)		

CNY, Chinese yuan; a: χ^2^, b: F, *p < 0.05. Class 1, Low-FoP Adaptive Type; Class 2, High-FoP Sustained Type.

### 3.2 LPA results of patients’ FoP-Q-SF levels

As summarized in Table 2, AIC, BIC, and ABIC values decreased progressively with the addition of profiles. The 2-class model had an entropy of 0.919 (> 0.80), and both LMR (*p* < 0.001) and BLRT (*p* < 0.001) were significant. The average posterior probabilities for the two classes were both 0.98, indicating superior fit compared to the 1-class model. Thus, the 2-class model was selected as the optimal solution.

**Table 2:** Model Fit Indices for FoP Levels in Stroke Patients (*N* = 366).

Profile	Class 1	Class 2	Class 3	Class 4	Class 5
AIC	14681.002	12984.083	12353.584	12028.705	11833.032
BIC	14774.665	13128.481	12548.715	12274.570	12129.632
ABIC	14698.523	13011.095	12390.085	12074.696	11888.514
Entropy	NA	0.919	0.917	0.907	0.910
LMR (*P*)	NA	<0.001	0.152	0.051	0.085
BLRT (*P*)	NA	<0.001	<0.001	<0.001	<0.001
Category probability	1.0	48.6/51.4	30.9/49.7/19.4	15.8/15.6/32.0/36.6	35.8/15.8/18.6/18.9/10.9

AIC, Akaike Information Criterion; BIC, Bayesian Information Criterion; ABIC, Adjusted Bayesian Information Criterion; LMR, Lo–Mendell–Rubin; FoP, fear of progression; BLRT, Bootstrap Likelihood Ratio Test; NA, No Application.

**Figure 1:**
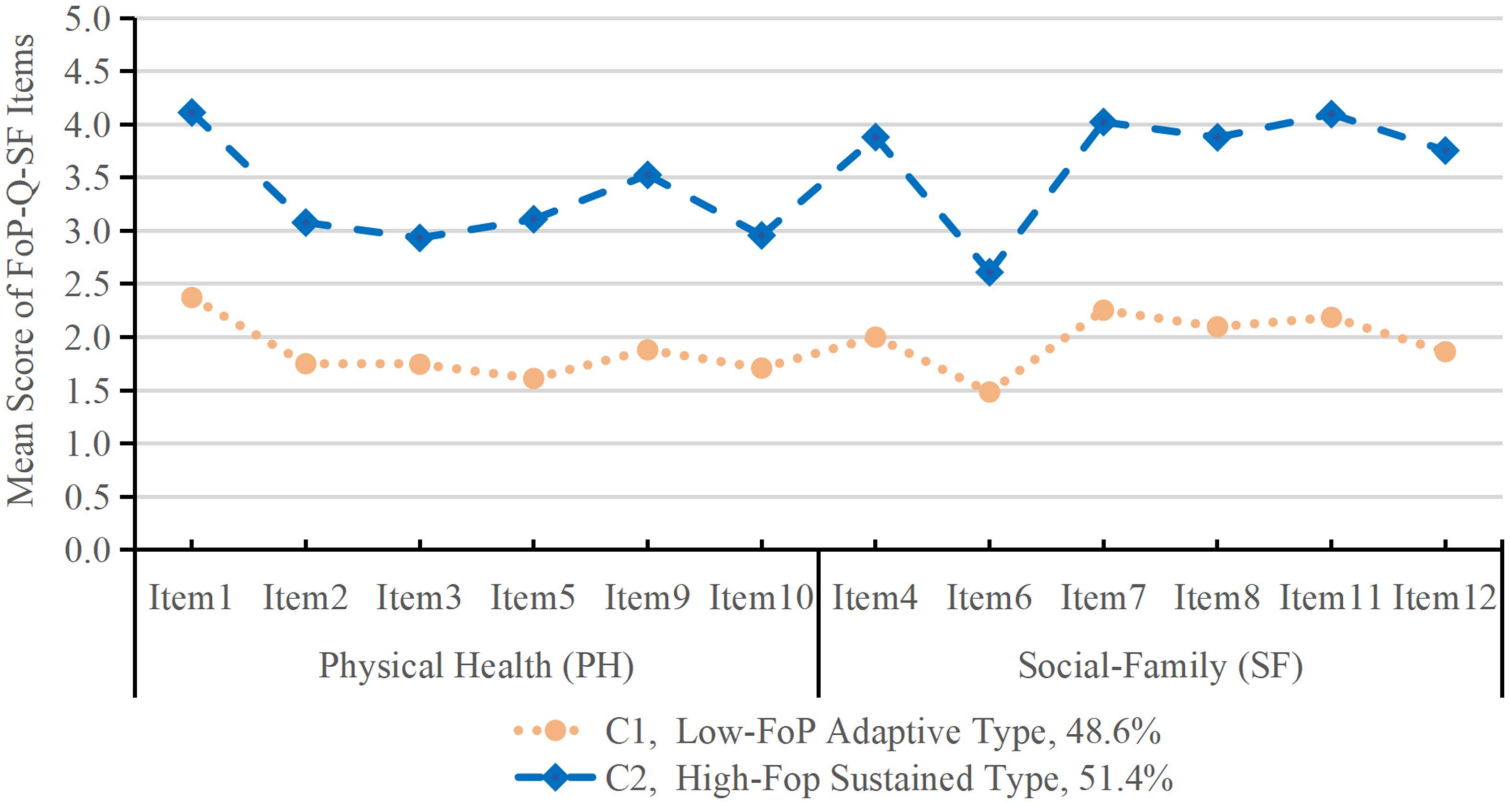
Two latent profiles of fear of progression in stroke patients.

The average posterior probabilities for C1 and C2 were 97.9 and 97.6%, respectively, confirming high classification accuracy. The mean scores of FoP-Q-SF items across subgroups are presented in Figure 1 and Table 3. Class 1 (C1, 48.6%) had overall lower item scores (1.91 ± 0.27) with a stable profile, labeled as “Low-FoP Adaptive Type.” Class 2 (C2, 51.4%) had significantly higher item scores (3.50 ± 0.53), labeled as “High-FoP Sustained Type.” Both subgroups scored highest on Item 1 (“I am afraid that my condition will worsen”), with C2 scoring 4.11 ± 0.81 and C1 scoring 2.37 ± 0.87.

**Table 3:** Comparison of FoP-Q-SF item scores across different FoP latent classes.

FoP-Q-SF items	C1 Mean (SD)	C2 Mean (SD)
F1 Being afraid of disease progression	2.37(0.87)	4.11(0.81)
F2 Being nervous prior to doctor’s appointment or periodic examinations	1.75(1.02)	3.08(1.22)
F3 Being afraid of pain	1.75(1.09)	2.93(1.26)
F4 Being afraid of becoming less productive at work	2.00(0.99)	3.88(1.03)
F5 Having physical symptoms (e.g., rapid heartbeat, stomach ache)	1.61(0.99)	3.11(1.22)
F6 Being afraid by the possibility that the children could contract the disease	1.48(1.05)	2.61(1.32)
F7 Being afraid of relying on strangers for activities of daily living	2.25(1.04)	4.02(1.03)
F8 Being afraid of no longer being able to pursue hobbies	2.10(0.94)	3.88(0.91)
F9 Being afraid of severe medical treatments in the course of the illness	1.88(0.99)	3.53(1.14)
F10 Worrying that medication could damage the body	1.71(1.04)	2.96(1.23)
F11 Worrying what will become of family if something happens to me	2.19(0.96)	4.10(0.89)
F12 Being afraid of not being able to work anymore	1.86(1.03)	3.75(1.12)

FoP-Q-SF, Fear of Progression Questionnaire-Short Form; SD, Standard Deviation; Class 1, Low-FoP Adaptive Type; Class 2, High-FoP Sustained Type.

### 3.3 Univariate analysis of factors associated with FoP latent classes

The two latent classes of FoP in stroke patients demonstrated statistically significant differences (*p <* 0.05) across the following variables: age, gender, monthly income, number of lesions, hypertension comorbidity, ADL scores, NIHSS scores, RRPSS scores, and perceived recurrence risk scores, avoidance, and resignation dimensions in MCMQ. The findings are presented in Tables 1, 4.

**Table 4:** Univariate analysis of FoP in stroke patients [*N =* 366, n (%)].

Variables	Class 1 n(%)	Class 2 n(%)	*χ^2^ /F*	*p*
NIHSS				
Mild stroke	149(83.7)	126(67.0)	13.768^a^	<0.001
Moderate stroke	27(15.2)	59(31.4)		
Severe stroke	2(1.1)	3(1.6)		
RRPSS	49.42 ± 10.08	53.70 ± 10.35	2.474^b^	<0.001
MCMQ				
Confrontation	16.22 ± 4.50	16.89 ± 4.65	0.007^b^	0.163
Avoidance	12.53 ± 2.30	13.14 ± 2.65	1.058^b^	0.020^*^
Resignation	7.93 ± 3.18	10.17 ± 3.62	4.373^b^	<0.001

a: χ^2^, b: F, *p < 0.05. NIHSS, National Institutes of Health Stroke Scale; RRPSS, Recurrence Risk Perception Scale for Patients with Stroke; MCMQ, Medical Coping Modes Questionnaire.

### 3.4 Factors influencing the latent class membership of FoP in stroke patients

The R3STEP method was used to analyze the influencing factors of FoP subgroup membership (reference group: Low-FoP Adaptive Type [C1]). The results showed that age 18–59 years, hypertension comorbidity, higher RRPSS score, MCMQ-confrontation, and MCMQ-avoidance were significant influencing factors (all *p* < 0.05). Gender, number of lesions, NIHSS score, and ADL score were not statistically significant (all *p* > 0.05). Detailed results are shown in Table 5.

**Table 5:** Regression analysis of FoP in stroke patients.

Variables	Class 2		95% CI	*p*
	B(SE)	OR		
Gender (Female = 0)				
Male	0.481(0.275)	1.617	0.944–2.773	0.081
Age (≥ 60 years = 0)				
18–59 years	−0.742(0.338)	0.476	0.245–0.924	0.028^*^
Household monthly income (< CNY 5000 = 0)				
≥ CNY 5000	0.489(0.385)	1.631	0.767–3.468	0.204
Number of lesions (Multiple = 0)				
Single	0.500(0.257)	1.649	0.996–2.728	0.052
Hypertension Comorbidity (No = 0)				
Yes	−0.911(0.270)	0.402	0.237–0.683	0.001^*^
RRPSS	−0.030(0.013)	0.971	0.946–0.995	0.022^*^
MCMQ				
Confrontation	−0.083(0.033)	0.920	0.863–0.982	0.011^*^
Avoidance	−0.228(0.049)	0.796	0.723–0.876	<0.001
Resignation	−0.076(0.049)	0.927	0.842–1.020	0.119

OR, odds ratio, 95% CI, 95% confidence interval, *p < 0.05. Reference group, Low-FoP Adaptive Type (Class 1); Class 2, High-FoP Sustained Type.

### 3.5 Results of difference tests for outcome variables between the two groups of participants

For stroke patients with different latent FoP profiles, statistically significant differences were observed in RRPSS, NIHSS, MCMQ-avoidance, and MCMQ-resignation (*p* < 0.05). Specifically, stroke patients in the High-FoP Sustained Type (C2) not only exhibited stronger subjective recurrence concerns ( $\overset{-}{x}$ = 53.812, SE = 0.772) and more severe neurological impairment ( $\overset{-}{x}$ = 4.096, SE = 0.319), but also tended to adopt negative coping styles such as avoidance ( $\overset{-}{x}$ = 10.227, SE = 0.270) and resignation ( $\overset{-}{x}$ = 13.171, SE = 0.198), as shown in Table 6.

**Table 6:** Analysis of differences in outcome variables among fop subgroups in stroke patients.

Variables	Class 1		Class 2		*χ^2^*	*p*
	$\overset{-}{x}$	SE	$\overset{-}{x}$	SE		
RRPSS	49.319	0.770	53.812	0.772	16.204	<0.001
NIHSS	2.781	0.260	4.096	0.319	9.737	0.002^*^
MCMQ						
Confrontation	16.210	0.344	16.894	0.347	1.857	0.171
Avoidance	7.882	0.243	10.227	0.270	39.744	<0.001
Resignation	12.514	0.176	13.171	0.198	5.864	0.015^*^

*p < 0.05. Class 1, Low-FoP Adaptive Type; Class 2, High-FoP Sustained Type.

## 4 Discussion

This study identified FoP heterogeneity via LPA using the FoP-Q-SF in stroke patients. We identified two distinct latent subgroups: “Low-FoP Adaptive Type” (C1: 48.6%) and “High-Fop Sustained Type” (C2, 51.4%). Furthermore, it further validated the correlates of FoP subgroups and between-class outcome differences by employing R3STEP for covariate analysis and BCH for outcome comparison. R3STEP and BCH analyzes confirmed that age 18–59 years, hypertension comorbidity, excessive recurrence risk perception, MCMQ-confrontation and MCMQ-avoidance were associated with high FoP. Additionally, high FoP patients had more severe neurological deficits (higher NIHSS score) and poorer adaptive coping abilities, highlighting the clinical significance of FoP stratification for stroke rehabilitation. These findings help identify high-risk populations early and develop personalized rehabilitation strategies to reduce FoP and improve outcomes.

In our study, the mean score of FoP was 32.67 ± 11.84, with 46.4% of patients scoring ≥ 34 points. This result is lower than those of studies on myocardial infarction and breast cancer patients (Wang X. et al., 2024; Zhuang et al., 2024), but significantly higher than colon cancer patients (Hu et al., 2024). Notably, the C2 subgroup accounted for 51.4% of the sample, indicating an overall elevated level of FoP among stroke patients, consistent with the findings of a meta-analysis by Yang et al. (2025). Participants in both subgroups scored relatively high on Item 1 (“I am afraid that my condition will worsen”). Although the C2 group scored significantly higher than the C1 group, this finding revealed a fundamental psychological vulnerability common to stroke patients. This divergence may stem from several factors, such as the following.

First, although recurrence rates in stroke patients have declined from 18 to 12% (Wang Y. et al., 2024), lingering uncertainty perpetuates disease progression anxiety. Moreover, persistent functional deficits, such as motor impairment and aphasia, act as tangible disease reminders, fueling immediate fear responses. Second, stroke rehabilitation entails prolonged, complex therapeutic regimens that potentially exacerbate adverse psychological outcomes (Lavados et al., 2021). Patients with stroke show relatively low psychological resilience (Wang Y. et al., 2024), and 31% with psychological stress (Almhdawi et al., 2021), which severely impedes rehabilitation and may further amplify FoP. This reminds healthcare providers and caregivers to focus not only on rehabilitation outcomes but also on active monitoring of psychological shifts and timely mental health interventions.

### 4.1 Pay attention to elderly populations

Our study confirmed that patients aged 18–59 years had a significantly lower risk of belonging to the High-FoP Sustained Type (C2) compared with those ≥ 60 years, which is consistent with the study by Liu Y. et al. (2025) and others on patients with hematologic malignancy. Elderly patients (≥ 60 years) generally require a higher level of care, often face greater physical function decline, longer rehabilitation cycles, and relatively modest rehabilitation expectations. Their fear stems not only from the pain of the illness itself but is also closely tied to concerns about their ability to fulfill family roles and responsibilities (Li et al., 2025). As a result, they are more prone to heightened concern over “disability and dependence resulting from disease progression.” In contrast, younger patients (18–59 years) typically have stronger rehabilitation potential and higher confidence in prognosis, even when shouldering multiple social roles (e.g., family breadwinners; Liu R. et al., 2025). This suggests that clinical practice should establish an age-stratified intervention model, prioritizing psychological support for elderly stroke patients to mitigate their elevated risk of sustained high FoP.

### 4.2 The impact of disease-related factors on FoP

An interesting result was found, which revealed that stroke patients with hypertension comorbidity were less likely to be classified as C2 than those without hypertension, which was inconsistent with the findings of another study (Song et al., 2021). This counterintuitive finding may be explained by the fact that patients with hypertension have long engaged in chronic disease management (e.g., regular medication, blood pressure monitoring), accumulating experience in symptom control and disease adaptation. Such experience enhances their tolerance for illness fluctuations and reduces excessive fear of stroke progression. This insight suggests that clinical teams can leverage hypertension management frameworks to integrate “disease adaptation training” into stroke rehabilitation, helping patients build confidence in illness control.

Our study showed a negative association between RRPSS scores and C2 membership, indicating that higher perceived recurrence risk is associated with a lower likelihood of sustained high FoP. Patients with higher perceived recurrence risk may proactively adopt health behaviors (e.g., adhering to medication, participating in rehabilitation) to reduce actual recurrence probability, and this sense of “controllability” mitigates excessive fear (Lei et al., 2025). Clinically, this implies that interventions should not merely aim to “reduce risk perception” but rather guide patients to transform risk awareness into constructive behaviors—for example, through personalized recurrence risk communication and coping skill training (Lin et al., 2021b).

Coping styles emerged as key correlates of FoP subgroups. R3STEP analysis showed that higher MCMQ confrontation scores and higher avoidance scores both reduced C2 membership risk, while BCH results confirmed that C2 patients had significantly higher resignation scores and avoidance scores. These findings highlight the dual nature of coping styles: (1) Confrontation, as an active coping strategy, reduces FoP by encouraging patients to address disease-related issues (e.g., consulting clinicians, participating in rehabilitation), which is consistent with another study (Wang et al., 2025). (2) Avoidance exhibits a complex “protective-burden” duality: it may temporarily buffer stress and alleviate acute anxiety in the short term (Chen et al., 2025); yet, persistent reliance on it can lead to the neglect of rehabilitation and an exacerbation of functional decline, ultimately trapping patients in a maladaptive cycle of “high fear - excessive avoidance - poor recovery.” (Thewes et al., 2016) Stroke patients may experience psychological distress due to various adverse outcomes, such as activity limitations, poor rehabilitation outcomes, social isolation, and poor functional recovery, which can lead them to adopt negative coping strategies, and inadvertently increases FoP (Weerasekara et al., 2024). Healthcare professionals should provide structured health education to guide patients in developing accurate disease understanding, dynamically monitor psychological changes, and empower patients to adopt adaptive coping strategies (e.g., transforming excessive avoidance into temporary stress management, strengthening confrontation skills) to mitigate FoP and enhance rehabilitation outcomes.

### 4.3 Limitations

This study has several limitations. First, the non-representative sampling method constrains the generalizability of the results. Future research should expand recruitment through multi-center studies across diverse regions to comprehensively characterize FoP heterogeneity among stroke patients. Second, the cross-sectional design cannot establish causal relationships or track long-term FoP trajectories. Longitudinal studies are needed to explore how FoP evolves during rehabilitation and its long-term impact on outcomes. Third, the MCMQ subscales for confrontation (Cronbach’s *α* = 0.64) and resignation (α = 0.67) had relatively low reliability, which may have led to attenuation bias in the results; future studies could use more reliable coping assessment tools or supplement item-total correlations for validation. Fourth, the examined determinants were limited to individual-level factors (sociodemographic, clinical, psychological). Future research should incorporate contextual factors (e.g., family support, healthcare system accessibility, social stigma) to develop a more holistic understanding of FoP.

### 4.4 Implications

To address diverse patient needs, clinical interventions should be customized according to the patient profile. Tailored health guidance and rehabilitation strategies are essential for different FoP subgroups. For patients with low FoP, key approaches include health education, psychological support, and mindfulness-based interventions. In contrast, for patients with high FoP, intensive personalized therapies, such as cognitive behavioral therapy (CBT) may be needed. Furthermore, integrating robust family and social support systems into stroke rehabilitation programs is critical for improving outcomes. This comprehensive approach effectively reduces FoP and enhances recovery outcomes.

## 5 Conclusion

This study identified two distinct latent profiles of FoP (Low-FoP Adaptive Type, High-FoP Sustained Type) among 366 stroke patients using LPA. R3STEP and BCH analyzes confirmed that younger age (18–59 years), hypertension comorbidity, excessive recurrence risk perception, MCMQ-confrontation, and MCMQ-avoidance were associated with high FoP. High FoP patients also had more severe neurological deficits and poorer adaptive coping abilities. These findings highlight significant FoP heterogeneity among stroke patients, emphasizing the need for early identification of high-risk individuals and targeted interventions. The results support the development of precision rehabilitation strategies to reduce FoP, enhance functional outcomes, and improve quality of life. Future research should use longitudinal and multi-center designs, incorporate contextual factors, and validate more reliable assessment tools to further explore FoP in stroke patients.

## Data Availability

The raw data supporting the conclusions of this article will be made available by the authors, without undue reservation.
